# Age-Related Differences in Doctor-Patient Interaction and Patient Satisfaction

**DOI:** 10.1155/2011/137492

**Published:** 2011-10-05

**Authors:** B. Mitchell Peck

**Affiliations:** Department of Sociology, University of Oklahoma, Norman, OK 73019, USA

## Abstract

*Background*. Relatively little is known about patient characteristics associated with doctor-patient interaction style and satisfaction with the medical visit. *Objective*. The primary study objectives are to assess: whether doctors interact in a more or less patient-centered style with elderly patients and whether patient age moderates the relationship between interaction style and satisfaction, that is, whether elderly patients are more or less satisfied with patient-centered medical encounters. *Methods*. We collected pre- and post-visit questionnaire data from 177 patients at a large family medicine clinic. We audiotaped the encounters between doctors and patients. Patient-centered interaction style was measured from coding from the audiotapes of the doctor-patient interactions. Patient satisfaction was measured using the Patient Satisfaction Questionnaire. *Results*. We found physicians were more likely to have patient-centered encounters with patients over age 65. We also found patient age moderated the association between interaction style and patient satisfaction: older patients were more satisfied with patient-centered encounters. *Conclusion*. Patient age is associated with style of interaction, which is, in turn, associated with patient satisfaction. Understanding the factors and processes by which doctors and patients interact has the potential to improve many facets of health care delivery.

## 1. Introduction

Clinicians, educators, researchers, and policy advocates generally agree that a more active and autonomous role for patients in the doctor-patient relationship is necessary to address health care needs [1]. Proponents of the patient-centered approach to health care delivery—in which patients' desires and expectations are incorporated into the medical decision-making process so that both physicians and patients contribute to the decision-making process [2]—suggest that eliciting and incorporating the patient perspective is associated with more favorable outcomes for patients. The evidence, however, is not conclusive. Some studies have shown that patients who are more active in the medical decision-making process report higher levels of satisfaction [3, 4], adherence to treatment regimens [5], and medical outcomes [6]. Other studies have failed to demonstrate a strong relationship between patient-centered care and patient outcomes [7, 8].

One plausible explanation for the mixed results is that the effect of the relationship between interaction style and outcomes may differ by patient characteristics (e.g., race, gender, age). Little empirical evidence exists about how patient characteristics might moderate the relationship between interaction style and outcomes [9, 10]. Furthermore, much of the evidence that does exist, does not examine actual doctor-patient interaction, but rather relies on preferences from vignettes and hypothetical scenarios [10–12].

Using data from audiotaped encounters between physicians and their patients, we examine the relationships among doctor-patient interaction style, patient satisfaction, and patient age. We focus on age for two reasons. First, age is relevant to both patient satisfaction and doctor-patient interaction. A recent review of patient satisfaction suggests that one of the most consistent findings is that age is positively associated with satisfaction with health care [13]. Age is also important in the conduct of the medical encounter, though the results are more mixed [11, 12, 14, 15]. Second, the aging of the American population means that the number of physician visits by older patients will continue to increase. Population estimates suggest that about one in five Americans will be age 65 or older in less than two decades [16].

Our primary study objectives are to assess: (1) whether doctors interact in a more or less patient-centered style with elderly patients and (2) whether patient age moderates the relationship between patient-centered interaction and satisfaction, that is, whether elderly patients are more or less satisfied with patient-centered interaction.

## 2. Materials and Methods

### 2.1. Study Participants

To address these issues, we use data collected from patients and physicians at a large family medicine practice over an 11-month period in 2007 and 2008. A total of 177 patients and 17 physicians are included in the analyses. The 17 physicians represent all the physicians in the clinic. We excluded other providers who treated patients, such as physician assistants and nurse practitioners. Study patients were recruited from the patient pool of participating physicians. Patients were identified and randomly selected from daily appointment schedules. The patients were approached while waiting to see their providers. To be eligible for the study, patients had to be 18 years old or older, speak and understand English, and had to have a scheduled appointment with the physician who provided their usual source of primary care.

There were approximately 3500 patient appointments at the clinic by participating physicians during the data collection period. We approached a relatively small portion of all patients (*n* = 271) because once a patient consented, the research staff stopped recruiting patients to begin data collection for the enrolled patient (e.g., conducting the previsit interview, setting up audio equipment). Generally, the interviewers did not begin recruiting new patients to participate until a previously enrolled patient was seeing his or her physician. Of the 271 patients approached, 177 (65 percent) consented and completed all three phases of data collection (previsit questionnaire, audio-taped medical encounter, postvisit questionnaire) with no missing data on the variables in the current analyses. The remaining 94 patients refused or did not provide consent (*n* = 35,13  percent), were ineligible (*n* = 8,3 percent) for one of the reasons listed above, or did not complete all phases of data collection (*n* = 51,19 percent). Common reasons patients did not complete data collection included being sent from the examination area directly to other areas of the clinic (e.g., X-rays, laboratory), audio equipment malfunction, and leaving while the interviewers were enrolling other patients. We tested for differences between patients who completed all phases of data collection (*n* = 177) and those who did not complete all phases or had missing data (*n* = 51). We compared the groups on status characteristic and social demographic variables. The largest difference was for patient race. White patients were slightly more likely to be included in the analyses, 63.8 versus 54.9 percent (*P* value =  .247). There were no statistically significant differences between the groups.

### 2.2. Data Collection

We obtained consent from physicians prior to recruiting patient participants. We obtained consent from patients on the day of their visit. After obtaining consent, trained interviewers administered a previsit questionnaire to assess patients' general demographic characteristics and information about the purpose for that day's visit. Patients completed a brief post-visit questionnaire administered by interviewers immediately after the doctor's visit to assess what occurred during the visit (tests, procedures, medications, etc.) and their satisfaction with the visit and physician.

The clinic visits were audiotaped and then coded by trained coders. The tapes were coded using a coding scheme similar to the Roter interaction analysis system (RIAS). The RIAS is a method of coding doctor-patient interaction during the medical visit [17]. It is one of the most commonly used methods for coding doctor and patient encounters [18]. 

Consistent with the RIAS and other analyses of doctor-patient encounters [19], we conceptualize two general categories of communication in medical encounters: socioemotional (affective) and task-focused elements of exchange. To this end, every utterance (whether a single statement or complete thought) expressed by both patients and providers was coded into mutually exclusive and exhaustive specific categories that reflect the broader socioemotional and task-focused classification. There are 15 specific categories of socioemotional exchange. Examples of socioemotional exchange include “personal remarks/social conversation” which are exchanges unrelated to the specific medical task, rather they are intended as friendly gestures or greetings (e.g., “Did you see the big game last night? It was great.”), “empathetic” statements intended to interpret, recognize, or name the other's emotional state (e.g., “You must be worried about this.”), and “self-disclosing” statements (by physician) that describe personal experiences that have medical or emotional relevance for the patient (e.g., “I had this same surgery; I felt 100 percent better afterward.”). The 19 specific categories of task-focused exchange include: “orienting statements” which tell the other person what is about to happen or what to expect (e.g., “I'm going to check your blood pressure.”), “giving medical information” which are statements that do not explicitly direct the other's behavior, rather they are statements of fact or opinion relating to the medical condition (e.g., “No matter what I eat, I feel bloated afterwards.”). A listing of all the socio-emotional and task-focused subcategories is presented in the Appendix.

The specific coding subcategories approximate the “three function model” of medical interviewing. The medical interview has been conceptualized in various ways. One of the most widely used is the so-called “three function model” [20, 21]. Functions refer to the general goals common to most medical encounters [22, 23]. The three functions are information gathering, developing and maintaining a therapeutic relationship, and communicating information [20, 21]. Categories relating to the medical tasks of the visit include: information giving, counseling, and question asking (both closed and open-ended) in the areas of medical condition, therapy, prevention, and lifestyle behaviors. Related to the affective, socioemotional aspects of the visit are categories of personal remarks, approval, laughter and joking, agreement, and statements of worry, support, legitimation, empathy, reassurance, concern, and partnership. The specific variables generated from the coding are the number of utterances in each category, as well as ratios of one category to another.

The coding approach is tailored to exchanges specific to the medical encounter in that coding categories reflect the content and context of typical dialogue between patients and doctors during medical exchanges. In addition, identification and classification of verbal events are coded directly from audiotapes, rather than transcripts. The use of audiotapes allows assessment of tonal qualities to determine the content of exchanges. A noted limitation of using audiotapes of medical encounters, however, is the inability to consider nonverbal modes of communication [19].

To code the audiotaped encounters, we created a coding sheet with operational definitions for the variables. The coders trained by coding practice encounters not used in the analyses. The research staff monitored the training sheets and provided additional training as necessary. Once the trainers were assured that the coders understood the coding categories and operational definitions, the audiotapes were coded.

Subjective interpretation by coders and coding variability is a possibility even with extensive training and coding guidelines. To minimize the effect of subjectivity and coder variability, we used multiple coders, who independently coded the tapes. During the coding process, we performed checks to ensure that the coders stayed within training guidelines. After coding was complete, we performed analyses to assess coder consistency. We evaluated interrater agreement by randomly selecting 10 percent of the audiotapes for double coding. We computed kappa statistics (*κ*) for a random selection of categorical variables. The kappa statistic evaluates the extent of agreement between two or more independent evaluations of a categorical variable and takes into account the extent of agreement that could be expected beyond chance alone [24]. We computed intraclass correlation coefficients (ICC) for numeric variables. The ICC is a measure of agreement between coders or raters used when observations are scaled on an interval or ratio scale of measurement [24].

In addition to categorizing each utterance by doctor and patient, the coders rated the overall affect of the encounter. The ratings are based on coders' overall impression of the affective content of dialogue between the doctor and patient. We examined the extent to which the coders agreed (kappa statistic) on the overall assessment of how dominant the physician was compared to the patient (on a 1–5 scale) and how warm and friendly the physician was toward the patient (1–5 scale). We also examined the extent to which the coders agreed (ICC) in their coding of the amount of biomedical discussion between the doctor and patient and the amount of closed-ended questions asked by the physician. Agreement between the coders was very good. The *κ* coefficients ranged from 0.92 to 0.98; the ICC scores ranged from 0.88 to 0.97.

### 2.3. Variables

Three variables are central to the analyses: patient age, type of medical encounter, and patient satisfaction. Each is discussed in turn.

#### 2.3.1. Patient Age

We measured age (in years) from patient self-reports on the previsit questionnaire. For the analyses, we collapsed the ages into a dichotomous variable indicating whether a patient was age 65 or older.

#### 2.3.2. Patient-Centered Interaction

To measure encounter type, we used codes from the audiotaped medical encounters. We used those codes that indicate specific control or influence in the encounter. We summed similar coding categories into aggregate measures. We then used cluster analysis to identify groups of cases (encounters) that were similar along the dimensions of influence and control in the encounter. We clustered the encounters on six aggregated variables, three of which measured patient communication patterns and three that measured physician communication patterns: biomedical information giving, psychosocial exchanges, and questions (both closed and open ended). Each variable is a ratio of all talk to minimize the effect of the length of the medical encounter. These three categories of variables describing interaction in the medical encounter—questions, biomedical information giving, and psychosocial talk (for both patients and physicians)—are often used to measure the dimensions of the encounter that reflect the patient-centered versus physician-centered continuum of interaction styles [25]. Additionally, these three sets of variables reflect the three functions of the medical interview: data gathering, patient education, and relationship building [20, 21]. The cluster analysis produced observations with two categories: physician-dominated and patient-centered encounters. The variable is coded as a binary outcome indicating a patient-centered encounter.

We considered alternative methods of measuring doctor-patient encounters to capture the complexity of the encounters. We examined a continuous-level measure that used a composite of the ratios of doctor and patient talk on the key variables discussed above. We also examined an ordinal measure that used coders' assessment of the level of physician dominance in the encounter. The analyses using the alternative measures (not presented) did not change the substantive findings. Because the findings were not substantively different and because doctor-patient encounters are typically conceptualized as dichotomies (e.g., physician versus patient-centered), we present findings using the binary outcome.

Table 1 shows how patient and physician-centered encounters differ on the six variables used to create the encounter type variable. The numbers are ratios and represent the number of statements or questions of a given type to the total amount of talk. The table shows that physician-dominated encounters are characterized by high levels of biomedical talk (25 percent of the encounter is the patient giving biomedical information) and relatively little psychosocial discussion by doctors or patients (18 and 12 percent, resp.). By contrast, the patient-centered encounters are characterized by lower relative levels of biomedical talk and higher levels of psychosocial discussion.

In addition to the variables representing the ratio to all talk in the encounter, two other variables are presented in Table 1 to highlight the differences in the types of encounters. These variables are physician verbal dominance and physician communication control. Physician verbal dominance is a straightforward measure of who talked more in the encounter. It is the ratio of all physician statements to all patient statements. A value greater than 1.0 indicates that physicians talked more than patients. A value less than 1.0 indicates that patients talked more than physicians. Physician communication control is a cumulative measure of the controlling statements and directives from the physician and patient. It was calculated relating physician verbal control (defined by physicians' questions and imperative orientations and patients' biomedical information giving) to patient verbal control (defined by patients' questions and directives and physicians' information giving and counseling, both biomedical and psychosocial). It, too, is a ratio of physician to patient statements. A value greater than 1.0 indicates that physicians made more directive statements (requiring patients to respond in some manner) and asked more questions (requiring patients to respond with information). A value less than 1.0 indicates that patients made more directive statements (requiring physicians to respond) and asked more questions (requiring physicians to respond by giving information). In general, physicians talk more than patients in an encounter.

In physician-dominated encounters, doctors talk about 55 percent more than patients, while in patient-centered encounters, doctors talk about 24 percent more than patients. More important than the amount of talk is the nature of the talk. It could be the case that physicians talk more, but are asking questions, building rapport, and engaging in psychosocial talk. The physician communication control ratios suggest that this is not the case. In physician-dominant encounters, doctors give directives and ask questions 1.8 times more than patients do. By contrast, in patient-centered encounters, doctors give directives and ask questions about 1.5 times more than patients.

#### 2.3.3. Patient Satisfaction

We assessed patient satisfaction using the patient satisfaction questionnaire (PSQ) [26]. The 10-item instrument focuses on the humanistic attributes and interpersonal and communication skills of the physician. The items relate to being friendly, showing interest, listening carefully, encouraging questions, communicating effectively, being respectful, using plain language, explaining problems, being truthful, and sharing decisions. Responses to each item were recorded using a 1 to 5 scale (poor, fair, good, very good, excellent), with a higher score indicating higher satisfaction. The PSQ has strong internal reliability (*α* = .92). We used the PSQ, rather than other patient satisfaction measures because of its known psychometric properties [27] and the availability of published data from other studies [28]. In addition, the PSQ is well suited for the present study because it is a rating of physician interpersonal skills that are largely the focus of patient-centered interaction. For the analyses, we created a dichotomous variable indicating high satisfaction (above the median) with physician interpersonal skill.

#### 2.3.4. Control Variables

We include several patient and physician-level covariates that may affect interaction type and patient satisfaction with the medical visit. The patient-level variables include patient status characteristic variables, gender, and race. We also control for education, income, and health status. All patient-level variables are self-reported.

Race was measured by asking patients the racial group with which they were most identified. For the present analyses, we use a binary measure of race: white and nonwhite. Patients who self-identified any race category other than white are classified as nonwhite. The omitted reference group in the analyses is nonwhite. Gender is a binary variable indicating whether the respondent is male or female. The reference category is female. Education is a measure of patient's highest level of school completed. The categories are less than high school, high school graduate (or GED), some college (but no degree), college graduate, and graduate degree. The omitted reference category is graduate degree. Income is personal income represented by categories of less than $20,000, $20,000 to $29,999, $30,000 to $39,999, $40,000 to $49,999, and $50,000 and above. The reference category in the analyses is $50,000 and above. Health status is measured using the 12-Item Short Form Health Survey (SF-12). The SF-12 is a generic measure of mental and physical health status for adults with all pathologies and diseases. The mental and physical composites are scored from 0–100; a higher score represents a higher level of health and functioning [29–31].

The physician-level control variables are gender, race, and years of medical practice. Like the patient-level measures, the physician-level variables are self-reported. Race is measured with the categories white and nonwhite. The reference group in the analyses is nonwhite. Gender is a binary variable indicating whether the physician is male or female. The reference group in the analyses is female. Years of medical practice are a measure of experience. We intended to include physician age as a covariate in the multivariate models. We did not include the variable, however, because it was highly correlated with years of medical practice (*r* = .96).

In addition to the patient and physician-level characteristics, we control for encounter characteristics related to the duration of the doctor-patient relationship. We include the number of visits between doctor and patient in the previous 12 months and whether the visit is the first encounter between the patient and physician. Both measures were collected from clinic records at the time of the interview.

### 2.4. Analysis

The outcomes of interest, physician-centered medical encounter, and patient satisfaction are binary variables. We, therefore, present odds ratios from binary logistic regression analyses. Because the data are clustered—patients clustered by physicians—the individual observations are not independent, potentially affecting estimates of the standard errors. We conducted all analyses using the Huber-White sandwich correction for nonindependent observations [32, 33].

## 3. Results

### 3.1. Sample Characteristics

Descriptive statistics of variables describing the study sample of patients are presented in Table 2. The median age of the patient sample was 60 years old. A little more than a third (37.9 percent) of the patients was age 65 or older. The sample included slightly more female (54.8 percent) than male (45.2 percent) patients. A majority of the patients were white (63.8 percent). The patients were mostly high-school educated or higher, with about a quarter (24.3 percent) college educated. The respondents reported a relatively low annual income. Almost half (45.2 percent) report an income less than $30,000. Only about one-fifth of respondents reported an income above $50,000. The patients had relatively low scores on the mental and physical composite scores of the SF-12 measure. Both scores are slightly lower than the national age-normed scores. One of the questions on the SF-12 asks patients to rate their health from excellent to poor. Fifty percent of patient respondents reported their health as fair or poor.

Characteristics of the 17 physicians in the study sample are presented in Table 3. Physicians were predominately male (58.8 percent) and overwhelmingly white (82.4 percent). The sample included three nonwhite physicians. The physicians' ages ranged from 33 to 54, with about half (52.9 percent) under the age of 40. A slight majority (52.9 percent) of the physicians had practiced medicine between 5 and 10 years. The remaining doctors were evenly split between those with less than 5 years experience (23.5 percent) and those with 11 or more years of medical practice (23.5 percent). The number of encounters for each physician ranged from 4 to 16 (median = 10). All physicians in the study attended medical school in the United States.

The demographic composition of the physicians in the study is similar to national averages in the U.S. Nationally, for example, about 70 percent of physicians are male [34]. In the current study, males comprise about 60 percent of the sample. For race, about 15 percent of physicians in the U.S. are nonwhite [34–36] compared to about 17 percent in the present study.

Table 4 presents variables that describe the medical encounters. There were few first-time visits (6.2 percent). Most encounters were between physicians and patients who had interacted in the past. Similarly, most doctors and patients (80.8 percent) had interacted at least once in the previous 12 months. Almost a quarter (22.6 percent) interacted three to five times in the previous 12 months. The length of visits ranged from 9.5 to almost 70 minutes, with a median of 25.2 minutes. The number of patient complaints or requests ranged from zero to five. The median number of requests was one. About one-third (33.9 percent) of encounters had no patient complaints or requests. Patient-centered encounters were the most frequently occurring type of encounter (54.8).

### 3.2. Physicians and Styles of Interaction

Before examining the effects of patient age on style of interaction and its possible moderating effects on patient satisfaction, we examined interaction style by physician. As noted above, patient-centered encounters were slightly more common than physician-centered encounters. Table 5 shows the number of encounters for each physician and the type of encounters by physician. The data show that most physicians had both patient and physician-centered encounters. Only 2 physicians (#6 and #10) had only one type of encounter. Both those physicians had only patient-centered encounters. It is worth noting that the two physicians had the fewest number of encounters in the sample (*n* = 6 and *n* = 4). While most physicians conducted encounters in both a patient and physician-centered manner, about three-fourths of the physicians (*n* = 13) tended to have a predominant style of interaction; that is, a physician had one style over the other in at least a 2 : 1 ratio (or 67 percent). Thirteen physicians conducted the same type of encounter more than 67 percent of the time. Nine of the 13 physicians tended to conduct patient-centered encounters, while four of the 13 tended to conduct physician-centered encounters.

### 3.3. Patient Age and Style of Interaction

The data in Table 5 show that there is considerable variability in the doctor-patient encounters. We now turn to an explicit examination of the first objective of the study: whether the variability in medical encounters is explained by patient age. More specifically, do doctors interact in a more or less patient-centered style with elderly patients? The unadjusted and adjusted odds ratios assessing the association between patient age and patient-centered interaction style are presented in Table 6. In the unadjusted model, patient age is associated with interaction style. Patients over the age of 65 are more likely to have a patient-centered encounter with their physician (OR = 1.34, *P *=  .041).

Controlling for patient, physician, and encounter characteristics slightly attenuates the association between patient age and interaction style (OR = 1.29, *P *=  .067). The direction of the association, however, remained the same. In the multivariate model, white patients and patients with higher mental health status were significantly more likely to have patient-centered encounters. Male physicians and white physicians were significantly less likely to have patient-centered encounters. Patients who were seeing their physician for the first time were less likely to have a patient-centered encounter.

### 3.4. Interaction Style, Age, and Patient Satisfaction

The second objective of the study is to determine if patient age moderates the relationship between patient-centered interaction and satisfaction, that is, if elderly patients are more or less satisfied with patient-centered interaction. Table 7 presents a series of models to assess the relationships among interaction style, patient age, and patient satisfaction. The unadjusted model shows the association between interaction style and patient satisfaction. Patients in patient-centered medical encounters report significantly higher levels of satisfaction (OR = 1.76, *P *=  .007). The age-moderated model includes patient age and the interaction effect of patient age and interaction style. The model suggests that patient age moderates the relationship between interaction style and patient satisfaction. The association between interaction style and patient satisfaction is stronger for patients over the age of 65 (OR = 2.02, *P *=  .039).

The fully adjusted model includes patient age, interaction style, the interaction term, and control variables. The moderating effect was attenuated slightly but indicated the same relationship: the effect of interaction style on satisfaction was stronger for patients over the age of 65 (OR = 1.41, *P *=  .074). White patients and patients with higher mental health status were significantly more likely to report being satisfied. Patients with low levels of education were less likely to report being satisfied compared to patients with high levels of education. Patients in medical encounters with male physicians reported lower levels of satisfaction, while patients in encounters with white physicians reported higher levels of satisfaction. Patients in first encounters reported lower levels of satisfaction.

## 4. Discussion

Previous research suggests that empowering patients in the medical decision-making process leads to positive results for patients along several dimensions, such as satisfaction [3, 4], adherence to therapeutic regimens [5], and other medical outcomes [6]. Some studies even show positive results for physicians in terms of physician satisfaction [37] and fewer malpractice claims [38]. Other studies have failed to demonstrate a relationship between patient-centered care and patient outcomes. It is plausible that the mixed findings from previous research are partly due to differences in patient populations. There is relatively little empirical research that examines if patient characteristics are associated with patient-centered interactions or how patient characteristics might moderate the relationship between interaction style and outcomes. This study aimed to address these issues. We examined the association between patient satisfaction and age and interaction style to determine if physicians interact differently with elderly patients in terms of patient centeredness. We also sought to determine if patient age moderates the relationship between interaction style and patient satisfaction.

Patient age is important because of the increasing number of elderly patients due to demographic changes in the U.S. and because compared to some other patient characteristics such as gender and race, relatively little is known about age. There is reason, however, to suggest that age plays an important role in doctor-patient interaction. Previous research suggests that older patients have lower desire for involvement in medical decision making [39], perhaps because they learned the patient role at a time when traditional asymmetry was more prevalent [40], when patients were more deferential to physicians, and before the emergence of patient-centered care [41]. We examined patient satisfaction, rather than other potential patient outcomes, because patient satisfaction is important in its own right, but is also associated with many other patient outcomes [42].

Findings from our study suggest that doctors interact with patients differently depending on age and that age moderates the relationship between interaction style and patient satisfaction. We found that older patients were more likely than younger patients to interact with their physicians in ways consistent with patient-centered interaction. The results are in a different direction than might be expected based on previous findings [12, 14, 15]. Our results also suggest that age moderates the relationship between interaction style and patient satisfaction. Like some previous studies [4], we found that patients in patient-centered encounters reported higher levels of satisfaction with their physician and visit. Contrary to expectations based on previous findings [39–41], age moderated the association in a positive direction: patient satisfaction was higher among elderly patients in patient-centered encounters.

One plausible explanation for the somewhat unexpected results has to do with developmental age effects versus cohort effects. Previous research suggests that older patients may prefer or be more satisfied with a less patient-centered medical encounter. Researchers have assumed that the variation is the result of developmental differences related to age. In fact, the differences may be the product of cohort effects [43]. Early cohorts of elderly patients may have been more comfortable and more satisfied with more traditional, asymmetrical doctor-patient interactions [35, 36]. As patient-centered care becomes more common, older patients may no longer prefer more traditional doctor-patient interactions.

Another plausible explanation for the unexpected findings relates to the limitations of the study. The primary limitation of the present study is the sample of patients and physicians. Patient and physician participants are from a single practice. The nature of the practice or the geographical location of the practice is not necessarily representative of other medical encounters. Further, despite being selected for participation using random selection methods, the medical encounters are not necessarily representative of medical encounters at the study site. In addition, at least two of the study physicians list geriatric medicine as their board certified specialty. It is possible that patients of these physicians are particularly satisfied with their care. Preliminary analyses (not presented) suggest that there are few differences between patients of the geriatric physicians and the other physicians, though the number of patients in the study from these physicians is relatively small (*n* = 16).

In conclusion, our findings suggest that the patient characteristic age is important in the doctor-patient encounter. Patient age is associated with style of interaction, which is, in turn, associated with patient satisfaction. These findings point to additional potential research questions. We examined differences for patients age 65 and over versus those under the age of 65. Do these relationships hold for patients 75 and older or 55 and older? Future research should examine other cut points for age. Further research is also needed to disentangle the developmental versus cohort effect. Our study did not attempt to answer this question, but suggests, perhaps, that cohort is more important. Finally, the significance of age in the doctor-patient encounter suggests that it is necessary to investigate other patient and physician characteristics.

Health care is delivered in an exam room with doctors and patients interacting. As such, the interaction between doctors and patients has implications for individual and public health. Understanding the factors and processes by which doctors and patients interact has the potential to improve many facets of the health care system from costs to quality to outcomes.

## Figures and Tables

**Table 1:** Amount of talk in communication categories by style of doctor-patient interaction.

	Physician dominated (*n* = 80)	Patient centered (*n* = 97)	Overall (*n* = 177)
Ratio to all talk			
Patient biomedical information giving	0.25	0.18	0.22**
Patient psychosocial talk	0.12	0.16	0.14**
Patient question asking	0.01	0.02	0.01
Physician biomedical information giving	0.21	0.15	0.18**
Physician psychosocial talk	0.18	0.23	0.21**
Physician question asking	0.09	0.07	0.08*
Communication dominance			
Physician verbal dominance	1.55	1.24	1.30^†^
Physician communication control	1.79	1.54	1.65*

^†^ *P* <  .10, *P <  .05, **P <  .01, ***P <  .001.

**Table 2:** Characteristics of the patient sample (*n* = 177).

	*N*	%
Age		
Under 35	4	2.3
35–44	13	7.3
45–54	49	27.7
55–64	44	24.9
65–74	43	24.3
75 and Older	24	13.6
Gender		
Male	80	45.2
Female	97	54.8
Race		
White	113	63.8
Nonwhite	64	36.2
Education		
Less than high school	31	16.5
High school degree	36	20.3
Some college	67	37.9
College degree	24	13.6
Graduate school degree	19	10.7
Income		
Less than $20,000	35	19.8
$20,000–$29,999	45	25.4
$30,000–$39,999	32	18.1
$40,000–$49,999	27	15.3
$50,000 or more	38	21.5
Health status (SF-12), median		
Physical Health Composite Score	31.8	
Mental Health Composite Score	47.7	

**Table 3:** Characteristics of the physician sample (*n* = 17).

	*N*	%
Gender		
Male	10	58.8
Female	7	41.2
Race		
White	14	82.4
Nonwhite	3	17.6
Age		
Under 40	9	52.9
40 and above	8	47.1
Years of practice		
Less than 5	4	23.5
5–10	9	52.9
11 and above	4	23.5
Number of encounters, median	10	

**Table 4:** Characteristics of the medical encounters (*n* = 177).

	*N*	%
First encounter		
No	166	93.8
Yes	11	6.2
Number of encounters previous 12 Months		
0	34	19.2
1	30	16.9
2	73	41.2
3	17	9.6
4	12	6.8
5	11	6.2
Visit length in minutes		
Minimum	9.5	
Maximum	69.3	
Median	25.2	
Number of patient requests		
0	60	33.9
1	67	37.9
2	29	16.4
3	13	7.3
4	3	1.7
5 or more	5	2.9
Encounter type		
Patient-centered	97	54.8
Physician-centered	80	45.2

**Table 5:** Frequency and percent of encounter types by physician.

	Encounter type	
Physician	Physician-centered	Patient-centered
1	4 (30.8)	9 (69.2)
2	9 (60.0)	6 (40.0)
3	1 (10.0)	9 (90.0)
4	4 (30.8)	9 (69.2)
5	3 (33.3)	6 (66.7)
6	0 (0.0)	6 (100.0)
7	5 (31.3)	11 (68.8)
8	3 (27.3)	8 (72.7)
9	8 (72.7)	3 (27.3)
10	0 (0.0)	4 (100.0)
11	7 (43.8)	9 (56.3)
12	3 (50.0)	3 (50.0)
13	6 (75.0)	2 (25.0)
14	1 (16.7)	5 (83.3)
15	13 (92.9)	1 (7.1)
16	5 (50.0)	5 (50.0)
17	8 (88.9)	1 (11.1)

Total	80 (45.2)	97 (54.8)

Note: Percentages are in parentheses.

**Table 6:** Odds ratios from binary logistic regression predicting patient-centered interaction.

	Unadjusted	Adjusted
Patient age 65 or older	1.34*	1.29^†^
Patient characteristics		
Gender—male		0.92
Race—white		1.48*
Education—less than high school		1.12
Education—high school degree		1.13
Education—some college		1.33
Education—college degree		1.12
Income—less than $20,000		0.84
Income—$20–$29,999		1.04
Income—$30,000–$39,999		0.75
Income—$40,000–$49,999		0.92
SF12—physical health		1.01
SF12—mental health		1.04*
Physician characteristics		
Male		0.63^†^
White		0.37**
Years of practice		0.97
Encounter characteristics		
First visit		0.49^†^
Number of visits		1.10

Note: Coefficients are odds ratios. Standard errors are corrected using Huber-White sandwich matrix estimator that does not assume independence of cases within clusters. ^†^ *P *<  .10, *P <  .05, **P <  .01, ***P <  .001.

**Table 7:** Odds ratios from binary logistic regression predicting patient satisfaction.

	Unadjusted	Age moderated	Adjusted
Patient-centered interaction	1.76**	1.38*	1.27
Patient age over 65		0.47*	0.76
Patient-centered × age over 65		2.02*	1.41
Patient characteristics			
Gender—male			0.71
Race—white			1.69^†^
Education—less than high school			0.24*
Education—high school degree			0.65^†^
Education—some college			0.68
Education—college degree			1.33
Income—less than $20,000			1.13
Income—$20–$29,999			0.89
Income—$30,000–$39,999			0.97
Income—$40,000–$49,999			1.12
SF12—physical health			0.98
SF12—mental health			1.06**
Physician characteristics			
Male			0.61^†^
White			1.46^†^
Years of practice			0.99
Encounter characteristics			
First visit			0.50^†^
Number of visits			1.12

Note: Coefficients are odds ratios. Standard errors are corrected using Huber-White sandwich matrix estimator that does not assume independence of cases within clusters. ^†^ *P *<  .10, *P <  .05, **P <  .01, ***P <  .001.

## Appendix

### General and Specific Coding Categories

Socioemotional Exchange

Personal remarks/social conversation
Laughs/jokes
Shows approval
Gives compliment
Shows agreement or understanding
Backchannel responses (indicators of sustained interest, attentive listening)
Empathy
Shows concern or worry
Reassures, encourages
Legitimizes
Partnership
Self-Disclosure
Shows direct disapproval
Shows general criticism
Asks for reassurance.

Task-Focused Exchange

Transition words
Gives orientation/instructions
Paraphrase/check for understanding
Bid for repetition
Asks for understanding
Asks for opinion
Asks questions (closed-ended)—medical condition (open and closed-ended)
Asks questions (closed-ended)—therapeutic regimen (open and closed-ended)
Asks questions (closed-ended)—lifestyle (open and closed-ended)
Asks questions (closed-ended)—psychosocial feelings (open and closed-ended)
Asks questions (closed-ended)—other (open and closed-ended)
Gives information—medical condition
Gives information—therapeutic regimen
Gives information—lifestyle
Gives information—psychosocial
Gives information—other
Counsels or directs behavior—medical condition
Counsels or directs behavior—lifestyle/psychosocial
Requests for services or medication.
